# Spiral drawing analysis with a smart ink pen to identify Parkinson's disease fine motor deficits

**DOI:** 10.3389/fneur.2023.1093690

**Published:** 2023-02-10

**Authors:** Simone Toffoli, Francesca Lunardini, Monica Parati, Matteo Gallotta, Beatrice De Maria, Luca Longoni, Maria Elisabetta Dell'Anna, Simona Ferrante

**Affiliations:** ^1^Nearlab, Department of Electronics, Information and Bioengineering, Politecnico di Milano, Milano, Italy; ^2^Child Neuropsychiatry Unit, Department of Child Neurology, Fondazione IRCCS Istituto Neurologico Carlo Besta, Milano, Italy; ^3^Istituti Clinici Scientifici Maugeri IRCCS, Milano, Italy; ^4^Istituti Clinici Scientifici Maugeri IRCCS, Lissone, Italy

**Keywords:** smart ink pen, spiral analysis, Parkinson's disease, movement disorders, eHealth

## Abstract

**Introduction:**

Since the uptake of digitizers, quantitative spiral drawing assessment allowed gaining insight into motor impairments related to Parkinson's disease. However, the reduced naturalness of the gesture and the poor user-friendliness of the data acquisition hamper the adoption of such technologies in the clinical practice. To overcome such limitations, we present a novel smart ink pen for spiral drawing assessment, intending to better characterize Parkinson's disease motor symptoms. The device, used on paper as a normal pen, is enriched with motion and force sensors.

**Methods:**

Forty-five indicators were computed from spirals acquired from 29 Parkinsonian patients and 29 age-matched controls. We investigated between-group differences and correlations with clinical scores. We applied machine learning classification models to test the indicators ability to discriminate between groups, with a focus on model interpretability.

**Results:**

Compared to control, patients' drawings were characterized by reduced fluency and lower but more variable applied force, while tremor occurrence was reflected in kinematic spectral peaks selectively concentrated in the 4–7 Hz band. The indicators revealed aspects of the disease not captured by simple trace inspection, nor by the clinical scales, which, indeed, correlate moderately. The classification achieved 94.38% accuracy, with indicators related to fluency and power distribution emerging as the most important.

**Conclusion:**

Indicators were able to significantly identify Parkinson's disease motor symptoms. Our findings support the introduction of the smart ink pen as a time-efficient tool to juxtapose the clinical assessment with quantitative information, without changing the way the classical examination is performed.

## 1. Introduction

Handwriting analysis is considered a promising biomarker for PD assessment, as impairments in the gesture can occur before the onset of typical symptoms (1). For this reason, handwriting tasks performed on paper have been introduced, as they are simple and fast to perform (2). Archimedes' spiral can be a useful task in the clinical PD evaluation, since its shape can elicit tremor in upper limbs (2, 3). However, the evaluation concerns only the produced traces, without focusing on the underlying movements.

Since the uptake of digitizers able to capture the coordinates of the pen on the screen and the exerted pressure, a huge effort had been put into quantitative spiral analysis (4), resulting in a series of statistical and classification studies. Statistical studies aim at finding spiral-derived features characterizing the PD population and report a reduced velocity and applied pressure in the advanced stage of the disease (5), a decreased fluency during OFF state (6), and an impaired spatiotemporal drawing execution (7). Other work quantified the effect of medication in alleviating bradykinesia and tremor amplitude (8). Moderate correlations were found between UPDRS III and its sub-scores, and indicators measuring spatial irregularity (9), velocity variability (10), and pressure (5). Classification studies, starting from spiral-derived features, exploit machine learning (ML) algorithms to train models aiming at distinguishing PD patients from healthy controls (11–13). The best results, obtained from 62 PD patients and 15 controls, reached classification accuracies above 95% (14–16).

Although the examined literature highlights the potential of quantitative spiral assessment for the objective characterization of motor symptoms, some limitations hamper its adoption. These limits include the undermined naturalness of writing performed on the small and frictionless surface of a digitizer, leading to an altered execution (17). Most studies tried to restore the natural feeling using a sheet of paper over the device surface, but inaccuracies in pen lifts can arise due to the different pressure required by the two media (18). Moreover, the use of digitizers during clinical practice may not be straightforward and time-efficient, often requiring technical support of an operator.

To overcome such limits, this work aims at computing indicators from spirals drawn using an innovative smart ink pen (19–22) to: i) discriminate between PD patients and age-matched healthy controls using both statistical and ML methods; ii) assess the correlation with PD clinical scales. The pen is sensorized with an inertial measurement unit and a force sensor, and is designed to write on paper, allowing the quantitative assessment of handwriting tasks while preserving the gesture naturalness. The device works like a normal pen and could be employed in the clinical routine without requiring technical support or increasing the time spent for the visit.

## 2. Method

### 2.1. Smart ink pen

The smart ink pen (19) looks like a normal ink pen (height 147 mm, maximum diameter 14.65 mm weight 48 g), but it is enriched with a load cell connected to the pen tip—to record the force exerted on the writing surface—and with tri-axial accelerometers and gyroscopes—to detect motion and tremor. It includes a memory and a communication unit to store and transmit data through Bluetooth Low Energy. The sampling frequency is set to 50 Hz.

### 2.2. Participants

PD patients were enrolled by IRCCS Istituti Clinici Scientifici (ICS) Maugeri (Milan, Italy). Patients' inclusion criteria were:

- Age ≥ 18 years;
- PD diagnosis;
- Mini Mental State Examination (MMSE) ≥ 24;
- Absence of disorders impairing handwriting, other than PD.

Politecnico di Milano (Milan, Italy) recruited the age-matched control group, whose inclusion criteria were:

- Age ≥ 18 years;
- MMSE ≥ 24;
- No musculoskeletal, neurological, or cardiovascular disorders impairing handwriting.

Age, gender, handedness and MMSE were collected from both groups. Patients were evaluated through the UPDRS (23) and the Hoehn and Yahr (H&Y) scale (24). From the UPDRS, the Jankovic (25), Schiess (26) and Kang (27) scores for PD motor symptoms classification were derived. High scores correspond to a tremor dominant patient, low scores to an akinetic-rigid (26, 27), or affected by postural instability patient (25), while medium scores to a mixed (26, 27) or indeterminate (25) one. Participants signed an informed consent prior to participation in the study. The protocol was approved by the Ethical Boards of ICS Maugeri (2457 CE) and Politecnico di Milano (n. 10/2018), for the respective recruited group.

### 2.3. Acquisition protocol

Subjects were asked to trace a spiral with the smart ink pen, following a template printed on a sheet of paper, possibly avoiding lifting the pen. The operator asked the subject to perform the spiral drawing (maximum diameter 6 cm, five loops separated by 1.2 cm) starting from the center and following the template line. Subjects were sitting on a standard chair, in front of a desk (height 72 cm) and instructed to assume an ergonomic posture, the feet resting on the floor. Patients performed the tasks under the ON medication state and, given the asymmetry of PD symptoms especially in the early stage, both hands were tested. Controls performed the task only with the dominant hand. All subjects performed the test twice.

### 2.4. Data analysis

Data analysis was performed in Matlab^®^ R2021b for the indicator extraction and the statistical analysis, while ML algorithms were implemented in Python^®^ 3.8.10.

#### 2.4.1. Indicator extraction

This phase included the pre-processing of the raw signals, followed by the extraction of 45 relevant indicators, divided into 7 domains. The drawing product was not considered in the analysis. Kinematic signals were band-pass filtered (2–12 Hz) with a zero-phase, 4th-order Butterworth filter. The following subscripts will appear in the names of the indicators extracted from kinematics, to clarify which signal was used for the computation: “*_A*” for acceleration; “*_G*” for angular velocity; “*_G_filt*” for angular velocity filtered around the spectral peak, “_T” for tremor contribution [extracted from the acceleration through empirical mode decomposition (28)].

- *Kinematics*. Indicators in this domain reflect the spatiotemporal behavior of the drawing gesture. The time (*Execution_Time*) and the number of strokes (*Strokes_Num*) required to complete the drawing were computed. The average and the variation coefficient of the difference between consecutive extrema in angular velocity (*ConsPeakDiff_G_Avg* and *ConsPeakDiff_G_CV*) were extracted (29).
- *Force*. The force generated while drawing is a key feature of the disease (4, 30). The average and variation coefficient of the exerted force were extracted (*F_Avg and F_CV*). To measure force variability in terms of amplitude, we considered the overshoot (*F_OVS*) (12), which is the difference between maximum and median value, and the difference in consecutive peaks (*ConsPeakDiff_F_Avg* and *ConsPeakDiff_F_CV*) (29). We included the number of changes in force in the time unit (*NC_F*), which quantifies the oscillations in the force profile (12).
- *Smoothness*. These indicators are related to the fluency in the drawing execution, which is relevant in characterizing PD (30). The number of extrema in kinematic signals (*NC_A, NC_G*) was retained (12). The presence of high frequency movements was investigated through the Spectral Arc Length (*SPARC_G*) (31); this indicator was computed considering different thresholds (*10, 20, 30, 40, 45*, and *50*) referring to the percentage of the peak value considered for noise removal. The logarithmic dimensionless squared jerk was computed for acceleration (*LDLJ_A*) and angular velocity (*LDLJ_G*) (32).
- *Tilt*. The domain refers to the inclination angle of the pen and was quantified by its average (*Tilt_Avg*), variance (*Tilt_Var*), and coefficient of variation (*Tilt_CV*).
- *Frequency*. This domain comprises indicators that describe the frequency content of the kinematics. We computed the Power Spectral Density (PSD) estimates through Welch's method (window length = 500 samples; overlap = 50%; frequency resolution = 0.1 Hz). Given the PSD, the relative power was computed for both acceleration and angular velocity (*RPW_A* and *RPW_G*) in different frequency bands (0–2 Hz; 2–4 Hz; 4–7 Hz, and 8–12 Hz). For angular velocity, the maximum relative power in an interval around the peak was computed (*RPW_G_filt_max*). The mean harmonic power (*MHP_T*) (33) was implemented to measure the presence of high frequency components.
- *Amplitude*. These indicators measure the amplitude of kinematic signals in time and frequency. The root mean square of the acceleration (*RMS_A*) and angular velocity (*RMS_G*) was computed on 10-s segments of the signals. After filtering the angular velocity in an interval centered around the spectral peak, the RMS was applied on 1-s windows of the resulting signal and averaged for the extraction of the maximum value (*RMS_G_filt_max*) (34). The signal-to-noise ratio (*SNR_T*) was calculated as the ratio between the tremor signal filtered around the peak frequency, and the remaining noise. To assess how evident is the peak in the PSDs, we computed the relative outlier level (*Out_Lev_Rel_A* and *Out_Lev_Rel_G*) as the distance between the PSD peak and the PSD mean. The product between the relative outlier level and the PSD peak value produced the amplitude per outlier level (*AmpXOut_Lev_A* and *AmpXOut_Lev_G*) (35).
- *Regularity*. The domain measures tremor regularity. The occurrence of repetitive patterns in the tremor signal was quantified through the Approximate Entropy (*ApEn_T*) (36). Tremor predictability was also measured by Recurrence Rate (*RR_T*) and Determinism (*DET_T*) (37). The Tremor Stability Index, applied in Luft et al. (38) in postural activities, was adapted to the spiral drawing condition (*TSI_T*) to measure the frequency variability in tremor cycles. The angular velocity change rate was computed over 1-s windows and the maximum value was retained (*G_Rate_max*).

See Supplementary Table I for the summary of the indicators computed in the study.

#### 2.4.2. Statistical analysis

The statistical analysis was conducted with the two-fold aim of: i) finding the most suitable indicators for distinguishing the drawings executed with the dominant hand by patients and controls; ii) assessing which indicators correlate with clinical scales for the PD population. The mean of the indicators obtained in the two tests was considered in the analysis, to capture information not based on a single sample. For the first aim, after testing indicators normality with the Lilliefors test, the Unpaired *t*-test and the Mann-Whitney test were applied to normal and non-normal indicators, respectively. For the second aim, following previous studies (5, 9), correlation was assessed through Spearman's Rank Correlation Coefficient (RHO. |RHO| ≤ 0.3 weak; 0.3 < |RHO| < 0.7 moderate, |RHO| ≥ 0.7 strong) between the extracted indicators and a series of UPDRS-derived scores, the H&Y scale score, and the Jankovic, Schiess and Kang scores. The UPDRS-derived scores included the UPDRS II tremor item (nr.16); the total UPDRS III score; the UPDRS III resting tremor item (nr.20); the hands score, obtained as the sum of the following UPDRS III items: action or postural tremor of hands (nr.21), rigidity (nr.22), finger taps (nr.23), hand movements (nr.24) and rapid alternating movements of hands (nr.25).

The sample size was chosen according to (5), where significant correlations between indicators and clinical scales ranged from 0.356 to 0.650. We considered the mean value of these 2 correlation results (0.503), leading to a sample size of 29 (confidence level: 95%, power: 80%).

#### 2.4.3. Machine learning

As we were interested in identifying the most relevant indicators in the between-group discrimination, ML methods were employed. Classification models were trained to differentiate between patients and controls and model explainability techniques applied, to gain insight about the model reasoning.

Different models were tested. The logistic regression, acting as a reference, and three models based on decision trees: random forest, LightGBM (39) and Catboost (40). For each model, two subsets of indicators were evaluated: subset 1-all 45 indicators; subset 2-statistically different indicators in the between-group comparison. Given the reduced dimensionality of the available dataset, all trials were conducted employing the Leave-One-Out Cross Validation approach. The classifier performance was evaluated through Accuracy, f1 score, Recall and Precision. To gain a better understanding about the indicators importance and trend in the classification task, the Shapley Additive Explanation (SHAP) technique (41, 42) was applied on the model achieving the best performance. This allowed revealing the most sensitive indicators in the classification, thus increasing the model interpretability.

## 3. Results

### 3.1. Participants

Thirty participants per group were recruited. However, one patient and one control subject were excluded from the analysis as their traces were characterized by an excessive number of pen lifts (>20). Therefore, the analysis regarded the spirals drawn by 29 PD patients (gender: 14 M; handedness: 29 R; age: 72.52 ± 7.37 yo; MMSE: 27.77 ± 1.64; UPDRS III: 19.17 ± 7.67; years since onset: 7.34 ± 4.94) and 29 controls (gender: 11 M; handedness: 29 R; age: 72.28 ± 8.30 yo; MMSE: 28.21 ± 1.57). The statistical analysis did not reveal between-group differences in either age (*p* = 0.91) or MMSE score (*p* = 0.31). The demographic and clinical characteristics for all participants are reported in Supplementary Table II.

### 3.2. Statistical analysis

Table 1 summarizes the statistically significant results of the between-group comparison. The complete results are available in Supplementary Table III.

**Table 1:** Statistically significant results of the between-group comparison.

**Domain**	**Indicator**	**PD**	**Control**	***p*-value**
Kinematics	*Strokes_Num* [#]	1.5 (1.75)	3 (2.63)	0.005^*^
Force	*ConsPeakDiff_F_Avg* [arbitrary]	8.07 (5.17)	11.28 (6.56)	0.014^*^
	*NC_F* [#/s]	3.74 ± 0.68	3.09 ± 0.63	0.0004^***^
Smoothness	*NC_A* [#/s]	5.82 (0.51)	5.76 (0.32)	0.029^**^
	*SPARC_G_10* [a.u.]	−42.75 (41.99)	−23.65 (18.49)	0.0009^***^
	*SPARC_G_20* [a.u.]	−25.64 (44.72)	−12.62 (18.54)	0.005^**^
	*SPARC_G_30* [a.u.]	−15.79 (33.39)	−7.31 (7.70)	0.006^**^
	*SPARC_G_40* [a.u.]	−6.83 (18.82)	−3.85 (5.31)	0.011^*^
	*SPARC_G_45* [a.u.]	−5.35 (13.96)	−3.84 (4.70)	0.019^*^
	*SPARC_G_50* [a.u.]	−3.71 (11.85)	−3.00 (3.62)	0.005^**^
	*LDLJ_A* [a.u.]	−6.69 ± 0.99	−5.31 ± 1.15	< 10E-05^***^
	*LDLJ_G* [a.u.]	−12.32 ± 2.37	−9.26 ± 2.40	< 10E-05^***^
Frequency	*RPW_A_0-2* [a.u.]	0.52 ± 0.13	0.68 ± 0.14	< 10E-04^***^
	*RPW_A_2-4* [a.u.]	0.16 ± 0.05	0.13 ± 0.04	0.013^*^
	*RPW_A_4-7* [a.u.]	0.16 ± 0.05	0.10 ± 0.05	< 10E-04^***^
	*RPW_A_8-12* [a.u.]	0.15 ± 0.04	0.10 ± 0.05	< 10E-04^***^
	*RPW_G_2-4* [a.u.]	0.18 (0.09)	0.38 (0.38)	< 10E-05^***^
	*RPW_G_4-7* [a.u.]	0.51 ± 0.16	0.36 ± 0.10	< 10E-04^***^
	*RPW_G_8-12* [a.u.]	0.21 (0.16)	0.17 (0.13)	0.045^*^
Amplitude	*Out_Lev_Rel_G* [a.u.]	4.03 (1.42)	3.02 (0.74)	0.002^**^
	*AmpXOut_Lev_A* [a.u.]	0.0063 (0.0081)	0.0013 (0.0032)	0.0006^***^
	*AmpXOut_Lev_G* [a.u.]	0.056 (0.094)	0.015 (0.014)	0.0002^***^
Regularity	*RR_T* [a.u.]	0.28 (0.37)	0.69 (0.45)	0.0002^***^
	*DET_T* [a.u.]	0.64 (0.32)	0.94 (0.27)	0.0004^***^
	*TSI_T* [Hz]	4.84 ± 1.37	6.00 ± 1.76	0.007^**^

Indicators (measurement unit in square brackets, a.u. stands for dimensionless) trend in the 2 groups is reported: mean ± standard deviation for normal distribution, median (interquartile range) for nonnormal distribution. The *p*-value (^*^ < 0.05, ^**^ < 0.01, and ^***^ < 0.001) is reported in last column.

In the *Smoothness* domain, the reduced *SPARC* and *LDLJ* indicators for the PD group, together with an increased *NC_A*, reflected a less fluent drawing execution. This finding is in agreement with the dysgraphia manifestation associated to the disease (4, 30). As for *Frequency*, the occurrence of tremor in PD spirals was highlighted by a different power distribution in the PSD for both acceleration and angular velocity signals: patients were characterized by a higher relative power in the band associated with PD tremor (4–7 Hz), while lower proportions were observed in the lowest frequency bands (0–2 H z for acceleration, 2–4 Hz for angular velocity) (43, 44). In line with (35), indicators related to spectral peak deviation in the PSD (*Out_Lev_Rel_G, AmpXOut_Lev_A* and *AmpXOut_Lev_G*) revealed significantly more evident peaks in the 2–12 Hz band for the PD group.

**Figure 1:**
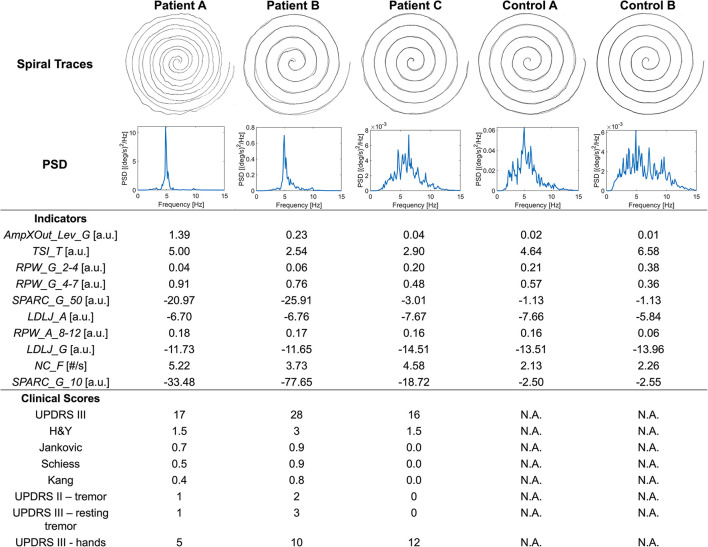
Spiral traces, angular velocity PSD, relevant indicators, and clinical scores for three patients (A–C) and two control subjects (A, B).

The importance of such domains is further explained by the examples in Figure 1. Patient A's spiral trace is affected by tremor and the occurrence of the symptom is well captured by the quantitative analysis. Indeed, the PSD of angular velocity is heavily concentrated around the peak, a fact that is represented by high values of *RPW_G_4-7* and *AmpXOut_Lev_G*. The reduced fluency during the execution is well captured by the *SPARC* indicators. Despite looking quite different with respect to Patient A spiral, Patient B spiral shows a similar behavior in terms of PSD and indicators. The less visible tremor in the trace generates lower values for *RPW_G_4-7* and *AmpXOut_Lev_G*, which are however above the central tendency of the PD group. Patient C presents the best-executed spiral among the three. However, *LDLJ_A* and *LDLJ_G* highlight a lack of smoothness in the drawing also in this case. Tremor is not evident from the trace, but its occurrence is detected by the analysis: the broader spectrum in the angular velocity with respect to patients A and B is translated into a lower *RPW_G_4-7*, yet approximately half of the total power.

Table 2 reports the correlations that resulted statistically significant. High values in Jankovic, Schiess and Kang scores, and in UPDRS III resting tremor score were associated with an increased *RPW_G_4-7* and *NC_A*. A reduced fluency in the drawing gesture was correlated with the overall impact of the disease: lower *SPARC* corresponded to high scores in H&Y (dominant hand) and UPDRS III (non-dominant hand).

**Table 2:** Correlation analysis results.

	**Dominant**			**Non-dominant**		
**Clinical score**	**Indicator**	**RHO**	***p*** **-value**	**Indicator**	**RHO**	***p*** **-value**
H&Y	*SPARC_20* [a.u.]	−0.40	0.033	*RPW_A_2-4* [a.u.]	0.41	0.030
	*SPARC_30* [a.u.]	−0.37	0.049			
Jankovic	*NC_A* [#/s]	0.56	0.002	*RPW_G_4-7* [a.u.]	0.45	0.017
	*RPW_G_4-7* [a.u.]	0.40	0.034			
	*ConsPeakDiff_G_Avg* [deg/s]	0.43	0.018			
Schiess	*NC_A* [#/s]	0.47	0.010	*RPW_G_4-7* [a.u.]	0.43	0.024
	*RPW_G_4-7*[a.u.]	0.39	0.037			
Kang	*NC_A* [#/s]	0.49	0.008	*RPW_G_4-7* [a.u.]	0.44	0.020
	*RPW_G_4-7* [a.u.]	0.37	0.045			
	*ConsPeakDiff_G_Avg* [deg/s]	0.41	0.029			
UPDRS II tremor	*RPW_G_4-7* [a.u.]	0.40	0.029	*RPW_G_4-7* [a.u.]	0.42	0.026
UPDRS III	*RPW_A_2-4* [a.u.]	0.37	0.049	*SPARC_30* [a.u.]	−0.39	0.043
				*SPARC_40* [a.u.]	−0.44	0.018
				*SPARC_45* [a.u.]	−0.45	0.015
				*SPARC_50* [a.u.]	−0.41	0.031
				*RPW_A_2-4* [a.u.]	0.50	0.006
UPDRS III–resting tremor	*NC_A* [#/s]	0.43	0.020	RPW_G_4-7 [a.u.]	0.50	0.007
	*RPW_G_4-7*[a.u.]	0.43	0.019	*AmpXOut_Lev_G* [a.u.]	0.38	0.048
	*RPW_G_8-12* [a.u.]	−0.37	0.048			
	*ConsPeakDiff_G_Avg* [deg/s]	0.37	0.046			
UPDRS III–hands	*F_CV* [a.u.]	0.41	0.027	*SPARC_40* [a.u.]	−0.43	0.022
				*SPARC_45* [a.u.]	−0.45	0.015
				*SPARC_50* [a.u.]	−0.42	0.028
				*MHP_T* [Log((mm/s^2^)^2^/Hz)]	0.40	0.033

For each clinical score, significantly correlated indicators are reported for the 2 hands with measurement unit, Spearman's RHO and *p*-value.

Although the significant correlation results, the correspondence between clinical scales and indicators was not always respected. For instance, considering Figure 1, patient B clinical scores are in line with indicators: the high UPDRS scores of tremor and resting tremor are reflected into increased *RPW_G_4-7* and *AmpXOut_Lev_G*. On the other hand, Patient A is reported with mild tremor and mild hand impairment, and Jankvoic-Schiess-Kang scores assign the patient to the postural instability/akinetic-rigid category. However, both trace and indicators show the occurrence of tremor (*RPW_G_4-7* and *AmpXOut_Lev_G*) and lack of smoothness in the drawing (*SPARC*).

### 3.3. Machine learning

**Figure 2:**
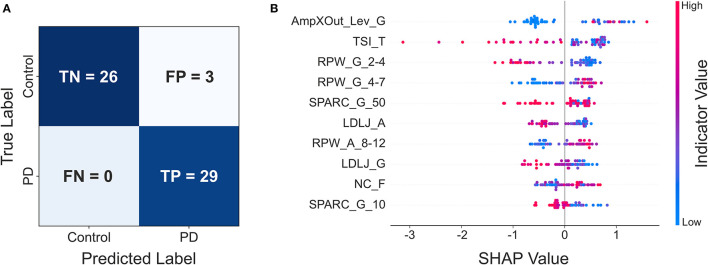
**(A)** Confusion matrix of the Catboost classifier trained with subset 2; **(B)** resulting SHAP plot.

Concerning ML classification with subset 1 (all indicators), the following performances were obtained: i) Logistic Regression, accuracy 84.48%, f1 score 84.21%, recall 82.76%, precision 85.71%; ii) Random Forest, accuracy 77.59%, f1 score 78.69%, recall 82.76%, precision 75.00%; iii) LightGBM, accuracy 89.65%, f1 score 90.00%, recall 93.10%, precision 87.10%; iv) Catboost, accuracy 87.93%, f1 score 87.72%, recall 86.21%, precision 89.28%. As for subset 2 (statistically significant indicators): i) Logistic Regression, accuracy 77.59%, f1 score 79.97%, recall 79.31%, precision 76.67%; ii) Random Forest, accuracy 79.31%, f1 score 79.99%, recall 82.76%, precision 77.42%; iii) LightGBM, accuracy 86.21%, f1 score 86.21%, recall 86.21%, precision 86.21%; iv) Catboost, accuracy 94.83%, f1 score 95.08%, recall 100%, precision 90.63%. The classification performances are summarized in Supplementary Table IV. Overall, the best performances were obtained by the Catboost model on subset 2, which allowed correctly classifying all PD patients, with only 3 misclassified controls (Figure 2A). The results of the SHAP analysis, performed on the Catboost model trained with subset 2, provided further insight about the way the different indicators impacted subjects' classification (Figure 2B). The plot presents the first ten indicators (according to SHAP results), in decreasing order of importance for the classifier decision. For each indicator, each point represents a subject and conveys two pieces of information: the SHAP value and the indicator value. The SHAP value is encoded by the horizontal position of the point: the more positive the SHAP value, the more the indicator pushes the classification of the subject toward the PD group, while negative values push it in the direction of the control group. The color represents the indicator value (red high, blue low).

This reveals, for example, that *AmpXOut_Lev_G* steered the most the classification of a subject toward the PD group (strongly positive SHAP values). Additionally, high (red) *AmpXOut_Lev_G* values are found only for positive SHAP values; this suggests that a clearly detectable peak in the angular velocity PSD leads the classification toward the PD group. A less variable tremor frequency (low *TSI_T*) pushed the prediction toward the PD group, as blue points are all located in the right portion of the graph. The power distribution represented another critical aspect for the differentiation: low *RPW_G_2-4* and high *RPW_G_4-7* pushed the classification toward the PD group, as they were associated with positive SHAP values. Low *SPARC_G_50, LDLJ_A, LDLJ_G* and *SPARC_G_10*, indicating the lack of fluency, were associated with a classification in the patient group (blue points only for positive SHAP value). High *NC_F* pushed the prediction toward the PD group, since red points are mainly concentrated in the right part of the plot.

The SHAP analysis allowed the investigation of the model reasoning, including gaining insight into the misclassifications. In Figure 1, Control A was one of the misclassified subjects (false positive) by the best model, while Control B was correctly assigned to the healthy group (true negative). Looking at Patient C (true positive) and Control A (false positive) traces and spectra, they both look similar. This similarity may explain why Control A was misclassified as PD. Indeed, looking at the *Frequency* indicators of Control A, *RPW_G_2-4* and *RPW_A_8_12* values are almost the same of Patient C, while *RPW_G_4-7* is even higher in Control A. Similarities are found in jerk-based indicators, highlighting a similar fluency. The *AmpXOut_Lev_G* value, although lower than in Patient C, highlights the presence of a more evident spectral peak with respect to the control group central tendency of 0.015. The *TSI_T* indicator reflects a stable tremor frequency, comparable to the one of the PD population. Altogether, these trends may be responsible for pushing the classification of Control A toward the PD group. Considering Control B, who was correctly assigned to the Control group, the trace is characterized by a good accuracy. The greater dispersion of its PSD over the entire frequency band, indicating the absence of relevant tremor components, is translated into comparable values of *RPW_G_2-4* and *RPW_G_4-7*, as well as in a reduced *AmpXOut_Lev_G* value. The highly variable tremor frequency (*TSI_T*), and the increased fluency of the acceleration signal (*LDLJ_A*) also underlie the correct classification.

## 4. Discussion

This work aimed at analyzing the spiral drawing execution of 29 PD patients and 29 age-matched controls, acquired with an innovative smart ink pen, to find the most suitable indicators in identifying and characterizing some disease motor symptoms.

A total of 45 indicators, divided into 7 domains, were extracted from the signals recorded by the pen inertial and force sensors, without information related to the spiral coordinates. Nevertheless, the outcome of the performed analysis was extremely good.

Significant between-group differences emerged in 25 indicators, with *Frequency* and *Smoothness* being the most relevant domains in the characterization of the disease. This is coherent with the spiral task, which is typically employed in pen-and-paper settings to elicit upper limb tremor and abnormal movement in neurological patients (2). Also the *Force* domain revealed trends in line with the literature, with patients applying a reduced and more variable force on the writing surface (45). The correlation analysis demonstrated that *Frequency* and *Smoothness* indicators are related with the patients' clinical scores. Great angular velocity power concentration in the 4–7 Hz tremor band and increased number of inversions in acceleration were correctly associated with high clinical scores assessing the occurrence of tremor (UPDRS II tremor, UPDRS III resting tremor, Jankovic, Schiess and Kang scores). The execution fluency decreased with increasing disease severity according to UPDRS III and H&Y scores. The correlation results were comparable with previous studies (5, 9) and, although significant, ranged from weak to moderate. We believe this does not indicate the indicators inaccuracy in quantifying the patients' symptoms, but rather reflects the well-known limitations of the clinical scales, including the low granularity of the assigned scores and the lack of separate scores for left and right side. Our hypothesis is supported by the identification of cases of mismatch between the clinical scores and the pen indicators, which were able to detect relevant alterations not visible from the spiral trace, nor from the clinical score. For instance, in Patient A, tremor and lack of fluency were detected in traces where their occurrence was not evident by visual inspection. These findings show how the use of the smart ink pen to perform clinical writing tests could be beneficial to complement the picture that emerges from the clinical examination with additional information related to the patient's conditions.

Considering classification, the performances of the Catboost model trained on subset 2 (only statistically significant indicators from the between-group analysis) were comparable to the best results found in the literature (14–16). But our focus was mostly on model explainability, a critical aspect in the path to the adoption of ML in healthcare: the understanding of the model decision-making is fundamental for clinicians (46). Yet, this aspect is poorly explored in the literature (14, 15), or provides results that are difficult to interpret (16). In our work, the SHAP analysis allowed identifying the most relevant indicators for the classification and gaining insight into the model misclassifications. Indeed, highly ranked indicators in the SHAP analysis exhibiting values similar to the PD group were responsible for the false positive cases.

Some limitations of the study can be pointed out. The sample size should be increased to further confirm the current results. In the recruitment, patients were clinically assessed by a single experienced rater; future work should study test-retest reliability of pen indicators compared to inter-rater agreement during clinical assessment. In future research, it would be interesting to study the differences between dominant and nondominant hands in both populations, as the between-group difference considered the dominant hand only. The conducted analysis should also be evaluated in patients at early stages of the disease—when patients' complaints cannot be clinically confirmed—or in preclinical stages, e.g., in PD genetic forms.

This work showed that the indicators extracted from the smart ink pen provide relevant information for the identification of PD motor symptoms. Such results support the use of the smart ink pen for PD spiral analysis in the clinical practice. Since the device looks like a normal ink pen and is used on simple paper, its introduction in the clinical examination would not change the way the spiral test is already performed, neither extend the duration of the visit. This point is crucial for adoption: given the increasingly limited time and resources in the healthcare systems, the smart ink pen represents a simple and time-efficient technology that transparently adapts to the clinical practice, supporting the graphomotor-based assessment with the identification of subtle but relevant patterns. Simplicity and transparent monitoring are two key requirements also for the remote health context. For this reason, the proposed device reveals important potential applications also in the remote patient assessment, with adequate frequency outside the clinical setting. The use of the device in both scenarios would allow improving and optimizing the treatment choice and result in improved patient's outcomes.

## Data availability statement

The raw data supporting the conclusions of this article will be made available by the authors, without undue reservation.

## Ethics statement

The studies involving human participants were reviewed and approved by Politecnico di Milano Ethical Committee (opinion n. 10/2018) and ICS Maugeri Ethical Committee (2547 CE). The patients/participants provided their written informed consent to participate in this study.

## Author contributions

ST: data analysis, manuscript writing, and manuscript final editing. FL: study design, data analysis, manuscript writing, and manuscript final editing. MP: study design, data acquisition, and manuscript revision. MG, BD, and LL: data acquisition and manuscript revision. MD: study design and manuscript revision. SF: acquisition of funding, study design, manuscript writing, and manuscript revision. All authors contributed to the article and approved the submitted version.
