# Supplementary material for: Spiral drawing analysis with a smart ink pen to identify Parkinson's disease fine motor deficits
Source: Front Neurol. 2023 Feb 10;14:1093690. doi: 10.3389/fneur.2023.1093690 (PMC9950270; doi:10.3389/fneur.2023.1093690)
Supplement: Supplementary file 1 [file Table_1.DOCX]

Supplementary Material

**Supplementary Table I:** indicators extracted in the study. For each indicator, the signal considered for the computation and the expected trend in the PD population, with respect to control subjects, is reported. For the detailed computation methodology, please refer to the references in the text. Prior to pre-processing, the force signal was used as reference to identify the beginning and the end of the execution. For acceleration and angular velocity, 3D means that the square root of the sum of squares of single axes was considered. a.u. = dimensionless unit.

| **Domain** | **Indicator** | **Signal** | **Trend in PD** |
| --- | --- | --- | --- |
| Kinematics | *Execution_Time* [s]  *Strokes_Num* [#]  *ConsPeakDiff_G_Avg* [deg/s]  *ConsPeakDiff_G_CV* [a.u.] | Timestamp  Force  Angular Velocity – Single Axis  Angular Velocity – Single Axis | Increase  Increase  Increase  Increase |
| Force | *F_Avg* [arbitrary]  *F_CV* [a.u.]  *F_OVS* [arbitrary]  *ConsPeakDiff_F_Avg* [arbitrary]  *ConsPeakDiff_F_CV* [a.u.]  *NC_F* [#/s] | Force  Force  Force  Force  Force  Force | Decrease  Decrease  Decrease  Decrease  Decrease  Increase |
| Smoothness | *NC_A [*#/s]  *NC_G* [#/s]  *SPARC_G_10* [a.u.]  *SPARC_G_20* [a.u.]  *SPARC_G_30* [a.u.]  *SPARC_G_40* [a.u.]  *SPARC_G_45* [a.u.]  *SPARC_G_50* [a.u.]  *LDLJ_A* [a.u.]  *LDLJ_G* [a.u.] | Acceleration – 3D  Angular Velocity – Single Axis  Angular Velocity – Single Axis  Angular Velocity – Single Axis  Angular Velocity – Single Axis  Angular Velocity – Single Axis  Angular Velocity – Single Axis  Angular Velocity – Single Axis  Acceleration – 3D  Angular Velocity – Single Axis | Increase  Increase  Decrease  Decrease  Decrease  Decrease  Decrease  Decrease  Decrease  Decrease |
| Tilt | *Tilt_Avg* [deg]  *Tilt_Var* [deg^2^]  *Tilt_CV* [a.u.] | Acc. and Ang. Vel. Single Axis  Acc. and Ang. Vel. Single Axis  Acc. and Ang. Vel. Single Axis | Decrease  Increase  Increase |
| Frequency | *RPW_A_0-2* [a.u.]  *RPW_A_2-4* [a.u.]  *RPW_A_4-7* [a.u.]  *RPW_A_8-12* [a.u.]  *RPW_G_2-4* [a.u.]  *RPW_G_4-7* [a.u.]  *RPW_G_8-12* [a.u.]  *RPW_G_filt_max* [a.u.]  *MHP_T* [Log((mm/s^2^)^2^/Hz)] | Acceleration – 3D  Acceleration – 3D  Acceleration – 3D  Acceleration – 3D  Angular Velocity – Single Axis  Angular Velocity – Single Axis  Angular Velocity – Single Axis  Angular Velocity – Single Axis  Acceleration – 3D | Decrease  Increase  Increase  Decrease  Decrease  Increase  Decrease  Increase  Increase |
| Amplitude | *RMS_A* [mm/s^2^]  *RMS_G* [deg/s]  *RMS_G_filt_max* [deg/s]  *SNR_T* [dB]  *Out_Lev_Rel_A* [a.u.]  *Out_Lev_Rel_G* [a.u.]  *AmpXOut_Lev_A* [a.u.]  *AmpXOut_Lev_G* [a.u.] | Acceleration – 3D  Angular Velocity – Single Axis  Angular Velocity – Single Axis  Acceleration – 3D  Acceleration – 3D  Angular Velocity – Single Axis  Acceleration – 3D  Angular Velocity – Single Axis | Decrease  Decrease  Increase  Increase  Increase  Increase  Increase  Increase |
| Regularity | *ApEn_T* [a.u.]  *RR_T* [a.u.]  *DET_T* [a.u.]  *TSI_T* [Hz]  *G_Rate_max* [a.u.] | Acceleration – 3D  Acceleration – 3D  Acceleration – 3D  Acceleration – 3D  Angular Velocity – Single Axis | Decrease  Increase  Increase  Decrease  Increase |

**Supplementary Table II:** demographic and clinical characteristics of recruited participants. MMSE is the Mini Mental State Examination score. The cut off for inclusion was MMSE score ≥ 24. For patients only, years since disease onset, UPDRS III and H&Y scores are reported. C = Control; PD = Patient; F = Female; M = Male.

| **Subject** | **Age** | **Gender** | **Handedness** | **MMSE** | **Years since disease onset** | **UPDRS III** | **H&Y** |
| --- | --- | --- | --- | --- | --- | --- | --- |
| C01  C02  C03  C04  C05  C06  C07  C08  C09  C10  C11  C12  C13  C14  C15  C16  C17  C18  C19  C20  C21  C22  C23  C24  C25  C26  C27  C28  C29  PD01  PD02  PD03  PD04  PD05  PD06  PD07  PD08  PD09  PD10  PD11  PD12  PD13  PD14  PD15  PD16  PD17  PD18  PD19  PD20  PD21  PD22  PD23  PD24  PD25  PD26  PD27  PD28  PD29 | 66  73  68  76  79  79  84  83  80  81  71  86  76  69  65  66  68  52  81  58  72  72  77  66  83  69  68  67  61  73  84  66  80  67  68  81  64  81  66  81  81  69  74  70  75  69  82  75  76  78  53  76  58  66  74  74  72  70 | M  M  F  F  F  F  F  F  F  F  M  M  F  F  F  F  M  F  M  M  F  M  F  M  F  M  F  M  F  M  M  F  M  M  M  M  M  F  F  F  F  M  F  F  M  M  F  M  F  M  F  F  F  M  F  F  M  F | Right  Right  Right  Right  Right  Right  Right  Right  Right  Right  Right  Right  Right  Right  Right  Right  Right  Right  Right  Right  Right  Right  Right  Right  Right  Right  Right  Right  Right  Right  Right  Right  Right  Right  Right  Right  Right  Right  Right  Right  Right  Right  Right  Right  Right  Right  Right  Right  Right  Right  Right  Right  Right  Right  Right  Right  Right  Right | 30  29  30  28  27  26  25  27  25  26  27  29  28  30  30  27  30  29  28  30  29  28  28  30  29  27  30  27  29  28  27  29  27.7  30  29  25  27.5  28  27  29  26  30  28  28  28  27  27  28  30  27  30  26  30  25  30  25  28  25 | N. A.  N. A.  N. A.  N. A.  N. A.  N. A.  N. A.  N. A.  N. A.  N. A.  N. A.  N. A.  N. A.  N. A.  N. A.  N. A.  N. A.  N. A.  N. A.  N. A.  N. A.  N. A.  N. A.  N. A.  N. A.  N. A.  N. A.  N. A.  N.A.  10  10  4  3  4  2  7  20  2  3  1  6  7  15  5  8  2  4  6  12  9  3  3  18  15  10  9  7  8 | N. A.  N. A.  N. A.  N. A.  N. A.  N. A.  N. A.  N. A.  N. A.  N. A.  N. A.  N. A.  N. A.  N. A.  N. A.  N. A.  N. A.  N. A.  N. A.  N. A.  N. A.  N. A.  N. A.  N. A.  N. A.  N. A.  N. A.  N. A.  N.A.  20  31  14  26  17  19  19  19  24  17  13  34  19  18  14  22  17  30  35  16  28  12  9  13  15  5  25  5  20 | N. A.  N. A.  N. A.  N. A.  N. A.  N. A.  N. A.  N. A.  N. A.  N. A.  N. A.  N. A.  N. A.  N. A.  N. A.  N. A.  N. A.  N. A.  N. A.  N. A.  N. A.  N. A.  N. A.  N. A.  N. A.  N. A.  N. A.  N. A.  N.A.  3  4  1.5  4  1.5  1.5  1.5  4  3  3  2  5  1.5  3  2  1.5  1.5  5  5  1.5  3  2  1  2  2  2  3  1  2 |

**Supplementary Table III:** results of the between-group comparison. Indicators (measurement unit in square brackets, a.u. stands for dimensionless) trend in the two groups is reported: mean ± standard deviation for normal distribution, median (interquartile range) for nonnormal distribution. The p-value (* < 0.05, ** < 0 .01, *** < 0.001) is reported in last column.

| **Domain** | **Indicator** | **PD** | **Control** | **p-value** |
| --- | --- | --- | --- | --- |
| Kinematics | *Execution_Time* [s]  *Strokes_Num* [#]  *ConsPeakDiff_G_Avg* [deg/s]  *ConsPeakDiff_G_CV* [a.u.] | 52.80 (34.11)  1.5 (1.75)  0.44 (0.37)  0.80 (0.13) | 61.75 (23.44)  3 (2.63)  0.39 (0.15)  0.85 (0.20) | 0.071  0.005^*^  0.093  0.181 |
| Force | *F_Avg* [arbitrary]  *F_CV* [a.u.]  *F_OVS* [arbitrary]  *ConsPeakDiff_F_Avg* [arbitrary]  *ConsPeakDiff_F_CV* [a.u.]  *NC_F* [#/s] | 134.77 (88.11)  0.22 ± 0.05  54.82 (33.07)  8.07 (5.17)  1.47 (0.23)  3.74 ± 0.68 | 155.45 (63.96)  0.20 ± 0.04  61.01 (35.43)  11.28 (6.56)  1.43 (0.21)  3.09 ± 0.63 | 0.099  0.215  0.014^*^  0.014^*^  0.514  0.0004^***^ |
| Smoothness | *NC_A* [#/s]  *NC_G* [#/s]  *SPARC_G_10* [a.u.]  *SPARC_G_20* [a.u.]  *SPARC_G_30* [a.u.]  *SPARC_G_40* [a.u.]  *SPARC_G_45* [a.u.]  *SPARC_G_50* [a.u.]  *LDLJ_A* [a.u.]  *LDLJ_G* [a.u.] | 5.82 (0.51)  24.97 ± 3.67  -42.75 (41.99)  -25.64 (44.72)  -15.79 (33.39)  -6.83 (18.82)  -5.35 (13.96)  -3.71 (11.85)  -6.69 ± 0.99  -12.32 ± 2.37 | 5.76 (0.32)  25.04 ± 2.78  -23.65 (18.49)  -12.62 (18.54)  -7.31 (7.70)  -3.85 (5.31)  -3.84 (4.70)  -3.00 (3.62)  -5.31 ± 1.15  -9.26 ± 2.40 | 0.029^**^  0.933  0.0009^***^  0.005^**^  0.006^**^  0.011^*^  0.019^*^  0.005^**^  <10E-05^***^  <10E-05^***^ |
| Tilt | *Tilt_Avg* [deg]  *Tilt_Var* [deg^2^]  *Tilt_CV* [a.u.] | 5.08 (6.49)  113.79 (241.91)  1.61 (0.85) | 4.56 (5.13)  133.10 (253.61)  1.99 (0.58) | 1  0.320  0.128 |
| Frequency | *RPW_A_0-2* [a.u.]  *RPW_A_2-4* [a.u.]  *RPW_A_4-7* [a.u.]  *RPW_A_8-12* [a.u.]  *RPW_G_2-4* [a.u.]  *RPW_G_4-7* [a.u.]  *RPW_G_8-12* [a.u.]  *RPW_G_filt_max* [a.u.]  *MHP_T* [Log((mm/s^2^)^2^/Hz)] | 0.52 ± 0.13  0.16 ± 0.05  0.16 ± 0.05  0.15 ± 0.04  0.18 (0.09)  0.51 ± 0.16  0.21 (0.16)  0.34 (0.18)  9.49 ± 1.19 | 0.68 ± 0.14  0.13 ± 0.04  0.10 ± 0.05  0.10 ± 0.05  0.38 (0.38)  0.36 ± 0.10  0.17 (0.13)  0.36 (0.09)  9.07 ± 1.23 | <10E-04^***^  0.013^*^  <10E-04^***^  <10E-04^***^  <10E-05^***^  <10E-04^***^  0.045^*^  0.852  0.191 |
| Amplitude | *RMS_A* [mm/s^2^]  *RMS_G* [deg/s]  *RMS_G_filt_max* [deg/s]  *SNR_T* [dB]  *Out_Lev_Rel_A* [a.u.]  *Out_Lev_Rel_G* [a.u.]  *AmpXOut_Lev_A* [a.u.]  *AmpXOut_Lev_G* [a.u.] | 1183.39 (768.72)  0.57 (0.69)  0.21 (0.30)  -4.40 ± 0.84  3.62 ± 0.53  4.03 (1.42)  0.0063 (0.0081)  0.056 (0.094) | 1374.79 (725.78)  0.63 (0.98)  0.22 (0.12)  -4.16 ± 1.43  3.74 ± 0.57  3.02 (0.74)  0.0013 (0.0032)  0.015 (0.014) | 0.270  0.276  0.109  0.440  0.398  0.002^**^  0.0006^***^  0.0002^***^ |
| Regularity | *ApEn_T* [a.u.]  *RR_T* [a.u.]  *DET_T* [a.u.]  *TSI_T* [Hz]  *G_Rate_max* [a.u.] | 1.65 (0.076)  0.28 (0.37)  0.64 (0.32)  4.84 ± 1.37  0.014 (0.042) | 1.61 (0.17)  0.69 (0.45)  0.94 (0.27)  6.00 ± 1.76  0.041 (0.061) | 0.131  0.0002^***^  0.0004^***^  0.007^**^  0.054 |

**Supplementary Table IV:** results of the classification models. For each model, accuracy, f1 score, recall and precision are reported, separately for each subset. Subset 1: all indicators. Subset 2: statistically significant indicators from the between group comparison. The best performance in each metric is highlighted in bold.

| **Subset** | **Model** | **Accuracy [%]** | **f1 score [%]** | | **Recall [%]** | | **Precision [%]** | |  |
| --- | --- | --- | --- | --- | --- | --- | --- | --- | --- |
| 1 | Logistic Regression  Random Forest  LightGBM  Catboost | 84.48  77.59  **89.65**  87.93 | 84.21  78.69  **90.00**  87.72 | | 82.76  82.76  **93.10**  86.21 | | 85.71  75.00  87.10  **89.28** | |  |
| 2 | Logistic Regression  Random Forest  LightGBM  Catboost | 77.59  79.31  86.21  **94.83** | | 79.97  79.99  86.21  **95.08** | | 79.31  82.76  86.21  **100** | | 76.67  77.42  86.21  **90.63** | |
